# Requirements for the scale up of a geriatric aftercare program: a qualitative interview study with stakeholders – findings from the GeRas project

**DOI:** 10.1186/s12877-026-07567-8

**Published:** 2026-04-30

**Authors:** Catharina Roth, Simone Maier, Leonie Maier, Anna Bülow, Martin Bongartz, Bastian Abel, Patrick Roigk, Kilian Rapp, Janine Peiter, Brigitte Metz, Gisela Büchele, Jürgen M. Bauer, Petra Benzinger, Michel Wensing

**Affiliations:** 1 Department of General Practice and Health Services Research, Heidelberg University Hospital, Heidelberg, Germany; 2 Geriatric Center, Medical Faculty Heidelberg, Heidelberg University, Heidelberg, Germany; 3 Department of Clinical Gerontology, Robert Bosch Hospital, Stuttgart, Germany; 4 Geriatric Center Karlsruhe, ViDia Christian Clinics Karlsruhe, Karlsruhe, Germany; 5 Institute of Epidemiology and Medical Biometry, Ulm University, Ulm, Germany; 6 Medical Faculty, Ruprecht-Karls-University Heidelberg, Heidelberg, Germany

**Keywords:** Geriatric rehabilitation, E-health, Telemedicine, Post in-house rehabilitation intervention, Transitional care

## Abstract

**Background:**

The increasing demand for geriatric health services, particularly in rehabilitation, has led to significant delays in the delivery of care across many regions of Germany. In response, Germany has introduced several initiatives, including innovative care models such as the telemedicine integrating multimodal, home-based geriatric aftercare programs GeRas that aimed at improving service efficiency and accessibility in geriatric rehabilitation. While such programs may increase geriatric health services capacity further exploration is needed to assess the scalability of such programs. Thus, the objective of this study was to obtain the perspective of key stakeholders (e.g., health services provider) on the requirements for scaling up of geriatric aftercare programs including a telemedicine component.

**Methods:**

In a qualitative interview study, 12 key stakeholders were interviewed to obtain their perspectives on the requirement for such geriatric aftercare programs, as well as their scalability, including the integration of telemedicine, taking the GeRas program as an example. The interview data were analysed using Qualitative Content Analysis according to Kuckartz.

**Results:**

A total of four main themes emerged from the qualitative content analysis. Theme two, including six subthemes, addressed the requirements for scalability of geriatric aftercare programs. Stakeholders emphasized the need to address financial requirements and establish transparent remuneration models for healthcare providers (1). Personnel requirements (2), and structural needs, including cross-sectional collaboration, were also highlighted (3). Additionally, the development and implementation of educational concepts for both providers and participants were discussed (4). Key requirements for a geriatric aftercare program (5) and the role of telemedicine (6) were also considered.

**Conclusion:**

Clear definitions of structural, personnel, and financial requirements, along with interprofessional collaboration, were essential for the scalability and success of geriatric aftercare programs. However, findings show that the scalability of these programs remains complex, yet essential.

**Trial registration:**

German Clinical Trials Register (DRKS00029559). Registered 5/10/2022.

**Supplementary Information:**

The online version contains supplementary material available at 10.1186/s12877-026-07567-8.

## Background

The demand for geriatric rehabilitation in Germany has increased in recent years due to the ageing population. As life expectancy is rising and the number of older adults is growing [1–4], there is a higher prevalence of age-related health issues, such as chronic diseases and functional decline [5]. Additionally, advances in healthcare allow more older individuals to survive serious health events, further increasing the need for specialised geriatric services, including geriatric rehabilitation [6, 7]. The growing demand for geriatric health services [8], including geriatric rehabilitation, has led to delays between discharge from acute medical treatment and admission to geriatric rehabilitation across Germany. In some regions, older patients face waiting times of three weeks or more before they can access the necessary rehabilitation treatments [4, 9], leading to a potentially delayed recovery [6, 10]. At the same time, the length of rehabilitation is restricted to three weeks, rather than being adapted to individual goals and resources. However, an application for an extension of the rehabilitation measures can be submitted if relevant goals have not been met. In the face of waiting lists, facilities are reluctant to apply for extensions, leaving health care professionals in a dilemma: choosing between unmet needs for those on the waiting list and those who have not fully achieved their rehabilitation goals.

In Germany, the distribution of geriatric rehabilitation clinics varies greatly by federal state and by region, with fewer facilities potentially available in rural areas but also in large cities such as Berlin and Hamburg [9–11]. Geriatric rehabilitation is largely organized as inpatient rehabilitation in clinics. Given the large catchment areas of these clinics, most of them cannot provide geriatric rehabilitation at home. The vast majority of patients are admitted following acute hospital treatment. Yet, admission to geriatric rehabilitation is also possible to treat functional decline due to chronic disabling conditions. Before starting geriatric rehabilitation, patients must apply for financing from their health insurance company via a physician who certifies the need for treatment. Geriatric rehabilitation facilities admit patients with confirmation of cost coverage according to their capacity. After discharge, therapeutic treatment is left to the discretion of the general practitioner (GP), while the organisation of nursing care has to be managed by the patients themselves or their families. Health insurances companies can provide organisational counselling upon request [9].

Geriatric rehabilitation clinics play a crucial role in ensuring the health and functioning of older adults, improving their mobility and quality of life. In Germany in 2023, there were approximately 1,000 rehabilitation clinics, covering various specialities, including geriatric rehabilitation. Around 170 of these clinics were dedicated to geriatric rehabilitation [9, 11]. While the number of over-75-year-old adults in Germany increased by more than 11% between 2015 and 2023, capacity in geriatric rehabilitation clinics has barely changed over the same period [12]. While perceived inadequate remuneration is one of the reasons for this lack of expansion, a growing shortage of qualified healthcare professionals has hampered the facilities’ ability to adapt their capacities in many places. To meet the demands of an ageing population, existing services may need to be modified, and new services have to be developed.

To address growing capacity shortages in geriatric rehabilitation, Germany has implemented several strategic measures. Legislative reforms, such as the *Intensive Care and Rehabilitation Strengthening Act (IPReG)* [13], have simplified access to rehabilitation services by limiting administrative barriers and standardising treatment duration [9]. National funding initiatives under the *Digital Healthcare Act and the Hospital Future Act* are enhancing digital infrastructure and cross-sectional collaboration [14]. Lastly, calls for more adequate reimbursement rates aim to stabilise the financial viability of rehabilitation facilities, ensuring long-term capacity and quality of care [15]. In addition, innovative care models are being implemented to promote continuity of care through transitional support and telemedicine-based follow-up procedures [16].

In Southern Germany, to address the growing demand for geriatric rehabilitation services and the lack of individualized length of treatment, a three-month, multimodal, home-based intervention program, known as the GeRas program, has been implemented across three study centres between October 2022 and October 2024. This innovative care model specifically targets older adults following discharge from inpatient geriatric rehabilitation, aiming to support continued recovery, prevent functional decline, and reduce readmission rates [17]. It offers a multiprofessional intervention, which can be delivered either traditionally through a limited number of home visits and telephone calls or by utilizing an eHealth system with tablet computers, combining home visits and video calls. It builds on self-exercise with motivational support. The primary goal of the GeRas program is to enhance functional capacity and social participation in older adults’ post-discharge. Additionally, it aims to improve coordination and collaboration between providers of healthcare services during the transition from inpatient geriatric rehabilitation to the outpatient setting via an eHealth system [17]. Designed specifically for geriatric patients receiving care at rehabilitation clinics in Germany, the GeRas program seeks to address the needs of this vulnerable population [17].

Initial results from the process evaluation, which was conducted alongside the implementation period of the GeRas program, indicate that participants and healthcare providers involved in the implementation and delivery found the program beneficial, with several advantages reported over usual care. However, results showed that the eHealth system was perceived as challenging by both participants and healthcare providers, and the digital infrastructure still needs to be improved to fully support the delivery of the program. Despite these challenges, the program’s components, including outpatient physical exercise, counselling, and continuous care after discharge, contributed to improvements in participants’ perceived well-being [18, 19].

Given the positive experiences of participants and therapists [18, 19] with the GeRas program, it appears advisable to extend its implementation, or that of similar multimodal geriatric aftercare programs, to other regions of Germany. To support this scaling-up process, it is essential to identify and understand the roles of relevant individuals and organisations currently involved or likely to be involved in the future. These include healthcare providers, policymakers, and other key stakeholders. Engaging stakeholders in the scaling-up process is crucial, as it is expected to promote acceptance, facilitate practical and needs-oriented planning, enhance implementation success, and contribute to long-term sustainability through shared ownership and responsibility.

The aim of stakeholder involvement is to foster their commitment and provide opportunities for active participation in the process. Stakeholder engagement may be supported by empirical research, using methods such as interviews or surveys [20]. Thus, this qualitative interview study aimed to obtain the perspective of key stakeholders (e.g., health services provider) on the requirements for scaling up of multimodal, home-based geriatric aftercare programs - such as the GeRas program including a telemedicine component.

## Method

### Study design

This exploratory qualitative interview study is part of the process evaluation of the GeRas project. The main study was a three-armed, assessor-blinded, randomized controlled trial designed to implement and evaluate the GeRas program at three study centres, the AGAPLESION BETHANIEN Hospital Heidelberg, the Robert Bosch Hospital Stuttgart, and ViDia Christian Clinics Karlsruhe in Germany [17]. The Department of General Practice and Health Services Research at University Hospital Heidelberg, Germany, was responsible for the process evaluation conducted alongside the main study. This interview study aimed to engage key stakeholders and explore their views on the potential scalability of geriatric aftercare programs such as GeRas.

### Participants

Stakeholders, including geriatricians, general practitioners (GPs), physical therapists, employees of health insurance companies, and social workers, from various regions of Germany, were invited to participate in semi-structured telephone interviews. Inclusion criteria were professional experiences in the field of geriatrics and the broadest possible inclusion of key stakeholders from different regions with different experiences. Eligible participants were at least 18 years old and not involved in the GeRas program. Participation was voluntary and compensated with a € 50 incentive. All stakeholders provided written informed consent before the interviews were conducted.

### Sampling and recruitment

A purposive sampling strategy [21] was used, and potential stakeholders were recruited through the personal networks of members of the GeRas project team. In the first step, stakeholders were approached by an individual personally known to them, who introduced the GeRas project and informed them about the forthcoming invitation to participate in the qualitative interview study. Stakeholders were then sent an email containing a brief description of the GeRas program, the objectives of the interview study, and a link to the published study protocol [17]. A member of the process evaluation team was copied (CC) on this email to facilitate subsequent coordination. Stakeholders who confirmed their willingness to participate via email were then contacted directly by a member of the process evaluation team to arrange an interview. To enhance response rates, follow-up reminder emails were sent, and recruitment progress was systematically monitored during weekly project coordination meetings.

### Interview guide

The interview guide was developed by a female health services researcher with a nursing background (CR) and a female research assistant with expertise in health services research and occupational therapy (LM).Two senior researchers from the coordinating team (MB (a male Sports Scientist) and PB (a female Geriatrician)) reviewed the initial version, and adjustments were made until consensus was reached. The open-ended questions focused on stakeholders’ experiences with geriatric aftercare programs, requirements for scalability of aftercare programs, implementation factors, and expectations. The leading questions were: (1) *What are your expectations of a home-based geriatric aftercare programs - such as the GeRas program including a telemedicine component* and (2) *In your opinion*,* how can a geriatric aftercare program*,* such as GeRas*,* contribute to successful aftercare?* A German and an English Version of the interview guide can be found as supplementary material 1.

### Data collection

Data was collected by the first author (CR) between July 2024 and October 2024 via semi-structured telephone interviews, all audio-recorded with participant consent. Socio-demographic data were also gathered to put the findings into context. Only the interviewer and interviewee were present during each interview. Interviews were pseudonymized and transcribed verbatim by a research assistant using the semantic transcription system [22]. Transcripts were not returned to participants for review, but accuracy was ensured by cross-checking transcripts against audio recordings. Data collection concluded once thematic saturation was reached, indicated by no new codes, subthemes, or themes emerging [23]. Data saturation was reached approximately after the eighth interview. All data are securely stored in accordance with data protection guidelines at the Department of General Practice and Health Services Research, University Hospital Heidelberg.

### Data analysis

The interview data were analysed using Qualitative Content Analysis based on Kuckartz [24]. After all interviews were transcribed verbatim, the first two authors (CR and SM (female Health Services Researcher)) familiarised themselves with the whole data set. In a next step, the first three interviews were inductively coded independently by CR and SM. The results were discussed, and an initial coding system was developed [24]. The following transcripts were then coded line-by-line by CR and SM independently. Each coded transcript was compared against the coding system in discussions, and disagreements regarding codes were resolved (involving a third senior researcher if necessary). The final coding system, including themes, subthemes, codes, and illustrative quotes, was discussed between CR and SM to ensure consensus. To support the analysis, MAXQDA version 2020.1.0, computer-assisted qualitative data management software, was used. Quotations included in this paper were translated into English (by CR, fluent in German and English) and adapted when needed, in order to maintain cultural meaning and checked for accuracy (by SM, fluent in German and English).

### Ethical approval

Research in this study was conducted in accordance with the Declaration of Helsinki. The study was reported according to the Consolidated Criteria for Reporting Qualitative Studies (COREQ) checklist for qualitative research [25] (supplementary material 3). The study has been approved by the local ethics committees at each study site (Heidelberg: Ethics Committee of the Medical Faculty of Heidelberg University [approval # S-395/2022]; Stuttgart & Karlsruhe: Ethics Committee of the State Medical Association Baden-Württemberg [approval # B-F-2022-057]).

### Quality of data management

Rigorous procedures were implemented to enhance the credibility of findings: (a) using more than one data coder during data analysis, (b) peer debriefing (qualitative research colloquium), (c) consensus discussion between the two coders, and, if necessary, a senior researcher, and (d) member checking with a senior researcher.

## Results

A total of 20 stakeholders were initially invited for an interview, 15 of whom were interested in participating. Due to scheduling difficulties, 12 interviews (response rate 60%) were conducted with members of four different professional groups by telephone between July 2024 and October 2024. A detailed description of the study population can be found in Table 1. Of the eight key stakeholders were initially invited but did not participate, three were GPs, four were geriatricians and one was an employee of a health insurance company. Reasons for non-participation included no response despite multiple contact requests or lake of time or interest.

**Table 1:** Description of the study population

Characteristics	*N* = 12 (100%)
Age in years (mean (SD))	44.9 (17.); Min 28 Max 66
Gender n (%)	
Identification as female	8 (66.7)
Identification as male	4 (33.3)
Federal State in Germany n (%)	
Baden-Württemberg	4 (33.3)
North Rhine-Westphalia	1 (8.3)
Lower Saxony	1 (8.3)
Rhineland-Palatinate	1 (8.3)
Bavaria	4 (33.3)
Hamburg	1 (8.3)
Profession n (%)	
Physical Therapists	3 (25.0)
Geriatrician	6 (50.0)
Sports Scientists	2 (16.7)
Employee of a Health Insurance Company	1 (8.3)
Type of facility n (%)	
Outpatient Physiotherapy Practice	3 (25.0)
Geriatric Clinic	7 (58.5)
Health Insurance Company	1 (8.3)
Research Centre	1 (8.3)
Work experience in Years (Mean, SD)	21.3 (9.7); Min 2 Max 30
Interview Duration (Median) in minutes	Range 32:29; Min 18:27 Max 42:50

A total of four main themes, each with several subthemes, emerged from the qualitative content analysis: (1) Perception of Current Care after Discharge from the Inpatient to Outpatient Geriatric Care, (2) Requirements for the Scalability of a multimodal, home-based Geriatric Aftercare Program, (3) Aims of Geriatric Aftercare Programs, and (4) Requirements for the Target Group. An overview of the main themes including the subthemes can be found as supplementary material 2.

Theme two, which focused on the scalability of the geriatric aftercare program, was specifically used to answer the research question of this article. Theme one, three, and four will probably be presented elsewhere and are not covered in this article, as this would go beyond its scope. An overview of theme two, including the related subthemes, is provided in Table 2.

**Table 2:** Overview of identified theme and subthemes addressing the research question

Theme	Definition
Requirements for the Scalability of a multimodal, home-based Geriatric Aftercare Program	This theme reflects the views and expectations of diverse stakeholders in Germany concerning the requirements for scaling up and sustaining of geriatric aftercare programs in general.

Subthemes	Definition
Structural Requirements	This subtheme outlines the structural requirements that need to be addressed to improve the scalability of geriatric aftercare programs.
Educational Concept	This subtheme emphasizes the importance of offering training to all participants receiving the program and providers of healthcare services involved in the delivery of the program.
Personnel Requirements and Network Building	This subtheme outlines the personnel requirements (1) and emphasizes the importance of network building (2) necessary to support and strengthen geriatric aftercare programs. Moreover, it deals with healthcare policy aspects (3).
Financial Requirements and Remuneration	This subtheme highlights the financial resources and remuneration models needed to ensure the scalability of geriatric aftercare programs.
Requirements for the Use of Telemedicine	This subtheme highlights the necessity of integrating telemedicine to enhance both the scalability and accessibility of geriatric aftercare programs.
Key Requirements of a Geriatric Aftercare Program	This subtheme illustrates the importance of implementing comprehensive, person-centered approaches within geriatric aftercare programs.

### Theme: requirements for the scalability of a multimodal, home-based geriatric aftercare program

This theme reflects the views and expectations of diverse stakeholders in Germany concerning the requirements for scaling up and sustaining geriatric aftercare programs.

#### Subtheme: structural requirements

This subtheme outlines the structural requirements that need to be addressed to improve the scalability of geriatric aftercare programs, including (1) bureaucratic hurdles, the (2) political support that is needed, the (3) discharge management that needs to be adapted, and (4) structural regional dependencies (e.g., location).

1. Bureaucratic Hurdles

Stakeholders emphasized that the responsibility for prescribing a geriatric aftercare program should be explicitly assigned and clearly defined to ensure a seamless transition of care for the patient. Additionally, they highlighted the critical role of communication among all players offering healthcare services. It was suggested that geriatric aftercare programs have the potential to enhance these professional interactions, thereby improving the overall quality of patient care. However, stakeholders also highlighted the operational heterogeneity among healthcare providers, which presents a barrier to the scalability and transferability of geriatric aftercare programs, such as GeRas, across different federal states and healthcare insurance systems within Germany.

*“How it is prescribed*,* how do I get it prescribed*,* does it work smoothly*,* do I have to submit an application? So I think it’s just the bureaucracy behind it*,* somehow overcoming that and getting it right in all the federal states (of Germany) with all the health insurance companies’ is a mammoth task.”* (CN1907).

*“Yes*,* I could imagine that it would be difficult to transfer this [GeRas] all over Germany*,* because every provider of healthcare services works differently.” (FW2407)*.

(2)  Political Support

Most stakeholders emphasized the importance of robust and transparent political support, as well as the early involvement of key stakeholders, such as health insurance companies, for the successful implementation and scaling of geriatric aftercare programs. They indicated that, without political reforms and a move away from the strict separation between inpatient and outpatient care, the implementation and expansion of geriatric aftercare programs will continue to face challenges.

*“‘And I simply believe that politicians need to do something to stabilise the healthcare system […].” (DFC2310)*.

*“[…] and it needs this counselling. It doesn’t work because the politicians are against it here in the region.[…]. They only care about their own counselling*,* where they can profit form it […]. And the politician who is primarily responsible for this says*,* I have someone who does that online […] a care counselling. An 80-year-old in online care counselling? So you can see the contrasts that exist. And you have to see if you have that. And I don’t know if there are actually social workers at the health insurance companies in Bavaria who provide this counselling. Normally*,* as the name suggests*,* care counselling is provided by nurses.” (KB2507)*.

(3)  Discharge Management

Stakeholders’ statements indicated that a structured discharge management from inpatient to outpatient geriatric care is essential for the sustainable implementation of a geriatric aftercare program. Given the heterogeneity in the providers of healthcare services, such as general practitioners (GPs), outpatient nursing services, therapists and clinics across Germany, adaptations are necessary in discharge procedures as well as the prescription and delivery of the aftercare program to ensure effective integration. However, stakeholders also identified that challenges extend beyond operational heterogeneity, highlighting staff shortages combined with a high demand for (geriatric) health services. Consequently, the feasibility of implementing structured discharge management alongside cross-sector communication and cooperation may be compromised.

*“Yes*,* that’s probably the first thing that comes to my mind […] it has to be linked somewhere to an inpatient institution*,* which have to take over the responsibility somehow or at least it has to be integrated into it. And I could imagine that it is very diverse in terms of how each healthcare institution is managed […].” (FW2407)*.

*“Well*,* I expect that the discharge management between the therapists will work smoothly in practice. In other words*,* contact should be made between the outpatient and inpatient therapists. There needs to be a connection.” (JB3007)*.

(4)  Structural Regional Dependencies

The interviewed stakeholders highlighted that regional factors, such as the distance between providers of healthcare services and program participants, may influence the implementation and scalability of geriatric aftercare programs. In some regions of Germany, geriatric healthcare service availability is limited, and geriatric aftercare programs have the potential to address and reduce these disparities. Furthermore, exercise and health programs should be tailored to regional characteristics and specifically accommodate individuals with limited mobility.

*“There is indeed a gap*,* which varies greatly from patient to patient*,* depending on where they live*,* the geographic area […].” (KB2507)*.

*“The travelling distance […] must be practicable*,* because otherwise it becomes difficult.” (KB2507)*.

#### Subtheme: educational concept

This subtheme emphasizes the importance of offering training to all participants receiving the program and providers of healthcare services involved in the delivery of the program. The following section, therefore, focuses on the training of (1) providers of healthcare services and (2) participants.

1. Training: Providers of Healthcare Services: Coordination, Realisation, Instructions for Participants, and Introduction to digital application.

From the stakeholders’ perspective, the development of a practical and targeted training concept for providers of healthcare services is essential. Each professional group involved requires a tailored training approach that addresses its specific roles and responsibilities. Interviewees emphasized the need for clear and structured interprofessional collaboration, noting that the training should define when each professional group becomes active and who holds decision-making authority at different stages. Beyond these coordination aspects, stakeholders also highlighted the necessity of an introduction to the digital application and guidance on how to effectively communicate with participants. Overall, the initial training concept should be intended to foster a sense of confidence and clarity among those involved in program implementation.

*„But it is definitely necessary for the individual professional groups to be clearly introduced to the manual or the apps. In other words*,* that such things have also been clearly trained*,* including which measurement instruments may need to be carried out*,* who carries them out*,* and who has to make which decisions and at what times they have to be made. And which group has to take which steps and plan when and how?“ (FW2407)*.

(2) Training Participants: Instructions for implementing the digital program.

The interviewed stakeholders emphasized the importance of providing training for participants, particularly regarding the use of the digital device and application. Several interviewees suggested that delivering this digital instruction during the inpatient stay would be beneficial, as it would allow for the immediate implementation of the program in the digital version following discharge. One stakeholder noted that such an approach could also help avoid the unnecessary use of resources in the home setting if the program ultimately proves to be unfeasible for the participant.

*„And then we thought about whether they could come round to the patients who are inpatients*,* explain and show them*,* just to see if it really works at home. Because before you hand out tablets and act*,* and then you have a patient who can’t do it at all.“ (DFC2310)*.

*„If we’re talking about tablets*,* we should also offer something technical in the clinic. Because otherwise something will fall out of the sky and it might be threatening or emotionally unfamiliar or whatever*,* right?“ (CB1810)*.

#### Subtheme: personnel requirements and network building

This subtheme outlines the personnel requirements (1) and emphasizes the importance of network building (2) necessary to support and strengthen geriatric aftercare programs. Moreover, it deals with healthcare policy aspects (3).

1. Personnel Requirements

Stakeholders highlighted the importance of including all professions involved in shaping healthcare for the older generation. Providers of healthcare services to be included encompass nursing professionals, physiotherapists, occupational and speech therapists, as well as social workers. From a medical perspective, involvement should extend beyond geriatricians to include GPs. According to the interviewed stakeholders, GPs could take on the role of coordinating the geriatric aftercare program. Furthermore, stakeholders emphasized that the participation of health insurance companies is crucial to facilitate the implementation and scaling-up processes. A few stakeholders pointed out that providers of healthcare services involved in the implementation and delivery of a geriatric aftercare program must be adequately motivated to actively participate in such an innovative initiative.

*“Exactly*,* I think it would be better to involve nursing professionals […]. Nurses should be involved*,* whether there is already an outpatient nursing service or nurses from the clinic*,* who can also assess the situation. Because nursing professionals have a more practical view*,* in my opinion.” (JB3007)*.

*“I believe it is very important to involve the health insurance companies […].” (DF2310)*.

*“Yes*,* I think it lives and dies with the motivation of the people who deliver the program. I think that’s very important. Who can be motivated?” (DF2310)*.

In general, according to the interviewees, it must be clear who takes responsibility for the program. The stakeholders suggested possible players: the clinic in which the participants were treated, GPs, the therapists, or the participants themselves.

*‘Who is the contact person*,* then? I think these things need to be clarified. Is it the clinic where the patient was previously treated*,* the patient themselves*,* or the GP?” (DFC2310)*.

(2)  Network Building

Concerning the scalability of geriatric aftercare programs, stakeholders considered network building among various providers of healthcare services essential. They emphasized the necessity of interprofessional communication and collaboration to enhance the quality of patient care, particularly in geriatrics. To facilitate such exchanges, the implementation of interprofessional case conferences, exemplified by the GeRas program, was proposed. Stakeholders particularly emphasized that cross-sectoral collaboration between involved providers and interprofessional exchange would contribute to enhancing healthcare quality in general; however, the presence of a dedicated coordinator remains indispensable. Additionally, some stakeholders regarded the establishment of a digital network encompassing multiple providers of healthcare services as a promising approach to support (cross-sectoral) interprofessional communication and collaboration.

*“Yes, I would also like to establish a network like this, […] or a quality circle. Maybe close to a geriatric centre, where you can say, okay, “I will share this now.” maybe we (different healthcare providers) will meet regularly and discuss the progress of the patients […].” (DFC2310)*

*“So I would be delighted if there was a network where therapists could work more closely together, however that might look. That the collaboration between inpatient and outpatient is strengthened. One option that I could imagine would be for clinics and practices to work together.” (JB3007)*

*“I also mentioned these interprofessional case conferences very briefly […]. I also think that these are very useful. I think an aftercare program should always include this. Because I think the central problem with all such programs is that the various healthcare providers do not actually communicate with each other and nobody knows what each other is doing […]. Yes, I think it can be a huge advantage.” (VH2507)*

(3)  Healthcare Policy Aspects

Stakeholders emphasized that in order to scale up and sustainably implement a geriatric aftercare program, aspects of healthcare policy must be considered, particularly given the essential interaction between inpatient and outpatient sectors. The transition of such a program into routine care requires careful planning, with the involvement of key stakeholders, such as general practitioners or physiotherapists, from the outset. Moreover, successful implementation relies on the active support of these stakeholders and requires a foundation grounded in robust empirical evidence.

*„Simply put*,* the first launch has to be right*,* yes? In other words*,* you really have to involve key stakeholders*,* and they really have to support it (the aftercare program)*,* yes? Of course*,* they have to be remunerated properly*,* that is clear*,* yes? But they have to really want to do it*,* they have to see a purpose […].“ (CB1810)*.

#### Subtheme: financial requirements and remuneration

This subtheme highlights the financial resources and remuneration models needed to ensure the scalability of geriatric aftercare programs. It includes (1) the need for sustainable financing of such programs through health insurance companies, and (2) appropriate remuneration for providers involved in delivering these services.

(1) Financing and (2) Remuneration.

The respondents’ statements clearly indicate that sustainable financing of geriatric aftercare programs through health insurance companies is essential for ensuring both their long-term viability and scalability. They stressed that without the involvement and financial commitment of health insurance companies, scaling up aftercare programs such as GeRas will not be feasible. Stakeholders emphasized that integrating geriatric aftercare programs into standard health insurance benefits would likely have a positive impact on scalability and sustainability. However, a recurring challenge raised by several stakeholders concerns the question of who should bear the financial responsibility for these programs. Furthermore, clarity regarding the remuneration of those delivering such programs is crucial.

*“I[…]*,* so one thing is always the financial aspect*,* if it is no longer part of a research project*,* who will finance the whole thing*,* and how will it be financed? I think that’s what’s crucial for patients. Unfortunately*,* for many patients*,* if it were on a self-pay basis*,* it would simply be problematic. So the question is always*,* how will it be covered financially?” (CN1907)*.

*“[…] get the health insurance companies on board and talk to them. Will they cover the costs*,* will they help to finance it?” (DFC2310)*

*“[…] and then you come back to the remuneration*, i.e.,* how is something like this reimbursed*,* and can it be charged via the classic prescription*,* or is a different concept perhaps necessary. So now you can prescribe everything separately*, i.e.,* is there a physiotherapy prescription*,* occupational therapy perhaps? Or should there actually be some kind of overall concept?” (FW2407)*.

#### Subtheme: requirements for the use of telemedicine

This subtheme highlights the necessity of integrating telemedicine to enhance both the scalability and accessibility of geriatric aftercare programs. It explores the potential (1) opportunities telemedicine offers for expanding care delivery, as well as the (2) challenges associated with its implementation in the context of geriatric aftercare. In addition, it discusses usability and digital sovereignty (3), including the need for hardware to participate in telemedicine components (4).

1. Perceived Opportunities

Stakeholders identified time savings as the primary advantage of a telemedicine approach for providers of healthcare services, particularly by reducing travel time to and from patients’ homes. Stakeholders suggested that such time efficiencies may help alleviate staff shortages. Furthermore, interviewed stakeholders emphasized increased accessibility as an additional benefit of telemedicine, enabling the inclusion of patients with significant mobility limitations or those unable to leave their homes altogether. Moreover, the adaptability and flexible delivery modes of telemedicine programs were highlighted as advantages. Integrating telemedicine into geriatric aftercare programs may also serve as a motivational factor, for instance, through the implementation of reward systems or opportunities for patients to engage in exchanges via chat platforms. Additionally, providing weekly summaries of achieved milestones or activities could further enhance patient engagement. Some stakeholders identified the implementation of a telemedicine approach as an opportunity to mitigate social isolation among older adults. Through telemedicine, older individuals could connect to others upon request, thereby facilitating the establishment of supportive peer networks.

*“On the one hand*,* for the physiotherapist*,* because he/she is simply much more flexible in terms of time management*,* you can do it anywhere (due to telemedicine) and you do not have to drive around.” (CN1907)*.

*“An advantage is accessibility*,* yes. I can just stay at home*,* I have my service there*,* I do not have to order a taxi*,* I do not need my relatives*,* I do not have to walk far or whatever*,* I have it right there (at home).” (VH2507)*.

*“[…] And of course the advantage of telemedicine is that I can integrate reminders or motivational messages*,* that I can also act in groups*,* so that I can communicate with others*,* and of course I can also get feedback on how much I have exercised or how much I have done today So there are a lot elements that speak in favour of changing behaviour.” (FW2407)*.

*“Yes*,* so the participants practically network with each other. Yes*,* exactly*,* so if they agree that you enable it […] that they’re in a chat […] or you actually make a live opportunity if they are very close together*,* in a smaller city […] so they can see that*.

*there is basically a network and that they can use it to socialise.” (AR2207)*.

(2) Perceived Challenges

In addition to the opportunities of the implementation of telemedicine in geriatric aftercare programs, several challenges were discussed. The primary challenge associated with the implementation of telemedicine, as emphasized by the interviewed stakeholders, was the lack of personal contact. They highlighted that, particularly for the older generation, face-to-face interaction with healthcare providers is essential to foster a sense of being well cared for and to maintain motivation.

*“Yes*,* I think so too. If I am understanding this correctly*,* there are video calls*,* there is a video consultation with a physiotherapist*,* which increases acceptance and motivation. I think that if you just play recorded material or a standard program*,* then the personal contact is simply missing. And that is very important in the generation that is now the target group.” (VH2507)*.

One solution, according to the stakeholders, is to integrate telemedicine as a supplement to in-person meetings. Specifically, an initial face-to-face meeting when introducing a digitally enhanced aftercare program. It was noted that interactions differ when people only know each other through an online format. Therefore, it is suggested to use telemedicine as a complementary approach, and even as an additional training tool.

*“The expectation would clearly be that the person you see on the screen is someone you’ve met before. Definitely. Because my own experience with teleconferences and so on is that you talk to each other very differently if you know the people beforehand.” (CB1810)*.

Furthermore, the protection of personal data was identified as a challenge by a few stakeholders in the integration of telemedicine within geriatric aftercare programs. Additionally, technical barriers such as an unstable internet connection or the complete absence of Wi-Fi infrastructure were reported. Another concern raised by interviewees pertained to the perceived lack of experience among the older population in handling technical devices, which may adversely affect the usability and effectiveness of telemedicine interventions.

*“Data protection! The most common barrier that has to be addressed […].” (*JB3007)

*“But of course that is a disadvantage*,* the technology can be a problem or that they simply do not understand it. Because they do not have signal (WLAN)*,* that aspect alone (is problematic). I think it is impossible for some people to connect their table to Wi-Fi at home. […] I think there are still some technical hurdles.” (CN1907)*.

(3) Usability and User Confidence

Stakeholders indicated that for telemedicine to be effectively implemented within geriatric aftercare programs, it must be accessible and user-friendly for older adults. Personalized (training) content should be easy to understand and easy to find. Additionally, it is essential to enable participation for people with visual impairments or fine motor skill difficulties when designing the program. Therefore, control elements and text should be sufficiently large for easy reading and tapping, and menu navigation should be designed in a simplified manner. Besides visual representation, verbal explanations are also considered helpful for this target group.

*“So if I can perhaps choose from a catalogue of exercises or even have my therapist change a program for me*,* then it’s tailored to me.” (VH2507)*.

*“It should be large and easy to see*,* and the control surface should be large and not so small […] For someone who no longer sees well*,* has lost fine motor skills or perhaps trembles*,* it should be designed to be large*,* clear*,* distinct*,* simple*,* and self-explanatory.” (BA0210)*.

Furthermore, obtaining technical support should be straightforward and free of obstacles to facilitate smooth adoption. Some stakeholders noted that the current generation aged 70 and above may have limited familiarity with technological devices and telemedicine; however, this is expected to evolve over time. Nevertheless, it is important not to underestimate or exclude this cohort based on assumptions regarding their technological proficiency or interest in telemedicine.

*“I think*,* older patients first have to learn how to use it (technical devices)*,* and many are not yet so good with the tablet […] but many just don’t know how to use it. So I do believe that this is a hurdle.” (AR2207)*.

(4)  Available Hardware

According to the interviewees, the prerequisite for implementing a telemedicine aftercare program is the availability of hardware and the necessary infrastructure, like having internet access or stable Wi-Fi. While the ‘bring your own device’ principle is emphasized, it is also acknowledged that devices must be provided to ensure the program is accessible to all. It can be assumed that not everyone in this generation has a digital device or access to the internet. On the other hand, it is seen as critical to involve another partner in the process who provides the devices due to the costs, or the financing of the provision of the devices should be clarified - on both sides: Participants and providers of healthcare services. If participants have their own devices, they are expected to be more familiar with them.

*“Of course*,* it must be ensured that the hardware is there*,* tablets are available.” (DFC2310)*

*“I think it’s most successful if you can do it entirely through your own system and devices*,* whether that’s a mobile phone or a tablet. Otherwise*,* a third party comes into play who practically has to provide the devices. In my opinion*,* this is a disadvantage because it increases costs and means that people have to get used to a new system.” (JB3007)*.

*“Financing would be required for a certain amount of technology.” (HO1308)*.

#### Subtheme: key requirements of a geriatric aftercare program

This subtheme illustrates the importance of implementing comprehensive, person-centered approaches within geriatric aftercare programs. The respondents’ statements clearly show that a holistic aftercare program is needed to meet the needs of those included in such programs. Therefore, the subtheme includes (1) a holistic program, (2) self-responsibility of the participants, and (3) the outcome of a geriatric aftercare program.

1. Holistic program

Stakeholders emphasized the need for continuous care following discharge from inpatient geriatric services, which they believed could be addressed through the implementation of a geriatric aftercare program. Interviewees expressed concern that post-discharge care is often lacking or that essential information is lost during transitions between inpatient and outpatient care settings. This was described as a trans-sectoral gap in care continuity. As a result, stakeholders stressed the importance of ensuring that the gains achieved during rehabilitation are sustained through seamless follow-up, to prevent rehospitalization and maintain the momentum of the training process.

Improving mobility through physical training was identified by stakeholders as one of the most critical components of geriatric aftercare, with particular emphasis on the importance of progression of intensity. However, this approach was seen to require home visits to effectively address individual needs. Stakeholders noted that home-based sessions enable therapists to tailor exercises to the participants’ specific abilities and integrate them into daily routines. Furthermore, potential challenges within the home environment can be identified and addressed directly during training sessions.

*“This means that mobility would be important for me at the forefront.“ (CB1810)*.

In addition to physical training, which some stakeholders viewed as the primary intervention, gerontopsychological counselling was regarded as highly relevant. Counselling on issues such as grief, despondency, and loneliness was specifically highlighted. Nevertheless, stakeholders pointed out that access to psychological support remains limited, with existing services often fragmented and poorly coordinated. Social counselling was also discussed positively, particularly when provided by a neutral body such as a health insurance company. Stakeholders emphasized that home visits offer valuable insight into an individual’s living conditions and facilitate the targeted and needs-based planning of care.

Stakeholders noted that approaches to cognitive training within occupational therapy, as well as nutritional management to prevent malnutrition, should be included in the aftercare program. Cognitive training was seen as part of occupational therapy or, alternatively, as a task that could be carried out by nursing staff. Nutritional management was also considered a component of nursing care, with one interviewee defining it as ensuring sufficient food intake to prevent malnutrition.

*“Of course*,* cognition can be supported to some extent by occupational therapy or nursing staff.” (OIO0909)*.

*“[…] nutrition*,* but I still consider that to be part of the nursing area*,* that care is taken to ensure nutrition in order to avoid malnutrition and the like.” (OIO0909)*.

Some stakeholders discussed the potential for transferring the program to other care settings. This included initiating the program in a discharge setting following inpatient hospitalization or continuing it after outpatient rehabilitation. One interviewee also suggested that implementation within long-term care facilities could be feasible.

*“Such an aftercare program in the nursing home*,* where this can then perhaps also be accompanied by the players there.” (VH2507)*.

(2)  Self-Responsibility of Participants

Participation in geriatric aftercare programs requires individuals to demonstrate self-responsibility and initiative. Understanding and appreciating self-responsibility should be part of a geriatric aftercare program, suggested one interviewee. This was regarded as the foundation for the participants’ self-efficacy.

*“The most important thing for me is to help older people understand and empathise with others*,* or to teach them that self-therapy is crucial. So*,* I have to do something myself.” (BA0210)*.

(3)  Outcome

Interviewees questioned whether a multi-week program could be successfully completed and sustained without ongoing support. At the same time, they emphasized the importance of documenting outcomes, particularly in the event of a potential inpatient readmission, to inform further treatment decisions. Overall, stakeholders agreed that personal support is essential to ensure completion of the program.

*„Of course*,* it would be super important to look up in my computer how GeRas or whoever did there. The patient did well in a follow-up program.“ (CB1810)*.

*„How long the effect lasts*,* i.e. when the direct support is no longer available*,* how sustainable it is.“ (HO1308)*.

## Discussion

Findings from this study demonstrate that in order to scale up and sustain geriatric aftercare programs, such as GeRas, structural, personnel, and financial requirements must be clearly defined and addressed well in advance. It is crucial to establish transparent remuneration models for providers delivering the program. Results from this study showed that key requirements of a geriatric aftercare program identified by various stakeholders were relatively consistent. Such a program should adopt a holistic approach, incorporating physical training and social and psychological counselling as demonstrated in a previous study by Roth et al. [19]. Additionally, stakeholders in this study indicated that participants in such programs are expected to demonstrate a degree of self-responsibility, internal motivation, and active engagement in their care process as demonstrated in an earlier study by Maier et al. [18]. Although telemedicine components were evaluated as beneficial by interviewees in this study, it was highlighted that the integration of telemedicine in general must be carefully planned and thoroughly implemented, particularly considering that the target population of geriatric aftercare programs may include a generation with potentially limited technological affinity. These findings are consistent with previous studies, which demonstrated that telemedicine can be a valuable tool but only if it is accessible and functional for all users [18, 19]. Additionally, the request for a blended care approach, which was also mentioned in previous studies, was highlighted [26, 27]. Moreover, findings of this study, along with those of previous research [18, 19], suggest that effective geriatric aftercare programs seem to rely heavily on building networks and improving the quality of interprofessional collaboration and communication across providers and sectors. It becomes apparent that the development and implementation of sustainable geriatric aftercare programs require a coordinated, multidisciplinary effort that aligns with the needs of both providers and patients, ensures structural feasibility and equitable access, and thoughtfully incorporates telemedicine as a supportive and inclusive component.

A report by the Federal Ministry for Education, Family Affairs, Senior Citizens, Women and Youth of Germany indicates that contrary to stereotypes, many older adults are capable of acquiring the necessary digital skills to navigate the digital world. However, this requires appropriate support structures at multiple levels [28]. The report states, that routine of use or user confidence enables older adults to act autonomously and competently in digital environments. While many possess or can acquire the necessary skills, targeted support is needed at individual, institutional, and societal levels. The findings of this report align with the results of this study and previous research [18, 19], on the implementation of geriatric aftercare programs, highlighting the importance of inclusive and well-supported digital solutions for older populations. Key measures include accessible training, user-centred technology design, and robust data protection frameworks. Thus, strengthening digital sovereignty in older adults requires both low-threshold learning opportunities and structural support to ensure meaningful and secure participation in geriatric aftercare programs including telemedicine components [28, 29]. Addressing these challenges is essential to empower older adults to participate not only telemedicine but fully in an increasingly digital society.

Regarding the requirements for implementing and scaling up such programs, it became clear that the stakeholders focused on the regulatory requirements and addressed the associated topics. They indicated that to ensure scale-up and sustainability, the development of a comprehensive educational framework for both providers and participants is essential. Additionally, the successful implementation of such a program, particularly one incorporating a digital component, requires the involvement of all stakeholders, including participants and their families [26, 30]. Moreover, mobile nursing service appears to play a pivotal role in this process, as highlighted by interviewees of this study. However, their role was not as clearly delineated as the role of community care nurses in the work by Preitschopf et al. [31]. This underscores the need for a regulatory framework to clearly define responsibilities and facilitate effective coordination between community-based care and inpatient geriatric rehabilitation.

This study has examined several key elements associated with the organisation and content of an outpatient geriatric rehabilitation, corroborating previous findings [30]. The core elements of an outpatient rehabilitation, i.e., a patient-oriented face-to-face treatment with an integrated eHealth solution, a structural collaboration, and including nursing care with educational approaches are also mentioned in another qualitative study [30, 32]. Stakeholders frequently mentioned the importance of counselling on assistive technology, emphasising that it can provide valuable support to people with disabilities, enabling them to remain in their homes for longer. In this context, occupational therapy was mentioned. However, the existing housing counselling structure offered by some municipalities was not mentioned in the interviews. One possible conclusion might be that such structures are not widely recognised, either due to a lack of knowledge or because counselling is not yet universally available [33]. Hence, its inclusion in geriatric aftercare programs should be considered and facilitate. Furthermore, speech and language therapists were mentioned by some interviewees as an important professional group within geriatric aftercare programs. However, their specific role remained unclear. It appears that their involvement is not considered necessary for all program participants but rather depends on individual needs and clinical indications. This underscores the necessity of clearly articulating individual care requirements and delineating professional responsibilities to support the effective scaling and long-term sustainability of such programs. Consequently, the integration of professionals from diverse disciplinary backgrounds is essential for the successful implementation, expansion, and maintenance of geriatric aftercare services.

The involvement of providers from diverse professional backgrounds was consistently emphasised across interviews as a key factor in the implementation and scale-up of geriatric aftercare programmes. In contrast, political support, specifically, the identification of relevant stakeholders and their respective roles in the scale-up process, was notably underexplored. This is particularly relevant given that evidence from German health-promotion initiatives indicates that successful scale-up efforts require the active engagement of policymakers, healthcare managers, and payers to establish appropriate legal frameworks, secure political commitment, and ensure sustainable financing [34]. The findings primarily reflect the perspectives of health service providers, who focused on practice-based challenges, while broader health system-level factors were often perceived as external and beyond their influence. This suggests a lack of cross-sectoral engagement, which may undermine the long-term sustainability and integration of geriatric aftercare programs into the wider healthcare system. Consistent with these findings, other studies have also highlighted the critical role of policymakers in enabling scale-up through dedicated advocacy, policy reform, and financial commitment [35]. It becomes apparent that stakeholder engagement is crucial throughout various implementation phases to shape priorities, adapt the program being implemented, guide methodological choices, and address implementation challenges [36]. However, results of this study highlight that appropriate stakeholders need to be identified prior to the implementation and the scale up process of geriatric aftercare programs. Medical Research Council and the National Institute for Health Research framework [36] can be used to guide the process.

### Strengths and limitations

The aim of this interview study was to obtain the perspective of key stakeholders (e.g., health services provider) on the requirements for scaling up of multimodal, home-based geriatric aftercare programs - such as the GeRas program including a telemedicine component. Thus, qualitative interviews were chosen as an appropriate method to gain in-depth insights into the perspectives of relevant target groups. To minimize bias and reduce the risk of content loss, data analysis followed standardized methodological procedures, and the COREQ checklist [25] was applied to guide the reporting of qualitative findings. Moreover, the inclusion of stakeholders from various regions of Germany allowed the findings to be interpreted within the context of the heterogeneity of the German healthcare system.

However, some limitations must be acknowledged. Although perceptions among the participating stakeholders (geriatricians and physiotherapists and one health insurance employee) were relatively consistent, the findings may not be generalizable to other healthcare providers who should also be involved in the implementation and scaling-up of geriatric aftercare programs, such as nursing professionals, occupational therapists, and speech and language therapists. Furthermore, policymakers, such as nursing home managers and representatives from general practitioner organizations, were not included in this study. Given the importance of political support for facilitating the scalability of aftercare programs, incorporating their perspectives on the requirements for scalability would have enriched the findings. While data saturation was reached, including a larger and more diverse group of key stakeholders from various professional backgrounds might have produced more varied results. Additionally, statements were translated from German into English, which may have resulted in subtle differences in meaning. Finally, the potential influence of social desirability bias on participant responses cannot be excluded.

## Conclusion

Stakeholders consistently emphasized the importance of a holistic, multimodal, home-based geriatric aftercare program that integrates physical training along with psychological counselling and aspects relevant to nursing care to ensure continuity of care after discharge. Telemedicine can play a valuable role in enhancing accessibility, particularly for individuals with limited mobility or those living in rural areas with restricted access to healthcare services. However, its implementation seems to be complex in the light of the regional heterogeneity within the healthcare system and technical challenges. To incorporate telerehabilitation, careful planning and implementation of telemedicine services remain important. To facilitate sustainability and scalability, it is essential to clearly define structural, personnel, and financial requirements and establish transparent remuneration models for healthcare providers involved in the delivery of the program. Moreover, strengthening interprofessional collaboration, including all healthcare providers involved in the care of older adults, particularly nursing staff, and enhancing cross-sectoral communication are crucial for the successful adoption and expansion of geriatric aftercare programs. In addition, educational concepts for providers and participants need to be developed. Overall, stakeholders seem convinced of the value of introducing geriatric aftercare programs; however, the implementation and scalability appear complex yet necessary, especially in light of capacity shortages in geriatric rehabilitation and the growing demand. While the results offer valuable insights, they cannot outline a clear or concrete process for their implementation.

## Supplementary Information

Supplementary Material 1.

Supplementary Material 2.

Supplementary Material 3.

Supplementary Material 4.

## Data Availability

The datasets generated and analysed during the current study are not publicly available due German data protection law but are available from the corresponding author on reasonable request.
