# Supplementary material for: Requirements for the scale up of a geriatric aftercare program: a qualitative interview study with stakeholders – findings from the GeRas project
Source: BMC Geriatr. 2026 Apr 30;26:612. doi: 10.1186/s12877-026-07567-8 (PMC13130819; doi:10.1186/s12877-026-07567-8)
Supplement: Supplementary file 1 — Supplementary Material 1. [file 12877_2026_7567_MOESM1_ESM.pdf]

Interview Guide (German and English) – Process Evaluation GeRas

German Version

Themenblock	Leitfragen	Aufrechterhaltungsfragen
**Einstiegsfrage**	**Vorab haben Sie Informationen bezüglich des GeRas Projektes erhalten. Das Ziel des GeRas Projektes ist es ein Nachsorgeprogramm mit Tablet oder Poster im Rahmen der geriatrischen Rehabilitation zu implementieren. Grob gesagt geht es darum, geriatrischen Patient:innen nach der stationären Reha ein Programm anzubieten, das den Übergang von der stationären Reha in das häusliche Umfeld erleichtern soll und Trainingserfolge, die in der stationären Reha erzielt wurden, zu festigen. Es handelt sich dabei um ein 12-wöchiges Nachsorgeprogramm mit unterschiedlichen Komponenten. Es zielt auf geriatrische Patient:innen ab, die in das häusliche Umfeld entlassen werden.<br>Kennen Sie ein ähnliches Nachsorgeprogramm bereits oder gibt es in Ihrer Umgebung etwas Ähnliches?**	
**Erfahrungen mit dem Klientel**	Aus Ihrer Erfahrung heraus, wie wird der Übergang derzeit zwischen der stationären rehabilitativen Behandlung und dem häuslichen Umfeld gestaltet?	• Wie läuft bei Ihnen die Versorgung geriatrischer Patient:innen aktuell im Anschluss an die stationäre Reha bzw. geriatrische Komplexbehandlung ab?<br>• Welche Erfahrungen haben Sie mit Reha-Nachsorge Programmen?<br>• Welche Situationen begegnen Ihnen in Ihrer alltäglichen Arbeit mit geriatrischen Patient:innen nach einem stationären Aufenthalt?
**Erwartungen an Nachsorgeprogramm**	Welche Erwartungen haben Sie an ein Nachsorgeprogramm wie GeRas?	• Wie würden Sie ein Nachsorgeprogramm für geriatrische Patient:innen gestalten?<br>○ Was wäre Ihnen besonders wichtig?<br>• Welche Voraussetzungen müssen Ihrer Erfahrung nach gegeben sein, damit ein Nachsorgeprogramm für geriatrische Patient:innen funktioniert?<br>• Welche Voraussetzungen müssen Ihrer Erfahrung nach gegeben sein, damit ein Nachsorgeprogramm für Versorgende funktioniert?<br>• Welche Rolle sehen Sie für Ihre Profession in einem Nachsorgeprogramm?<br>• Welche unterschiedlichen Stakeholder:innen sollten in die Gestaltung des Nachsorgeprogramms einbezogen werden?<br>• Wie bewerten Sie die ambulante Nachsorge, die aktuell im Rahmen der Regelversorgung nach geriatrischen Rehabilitationsmaßnahmen durchgeführt wird?<br>○ Wie würde die optimale Versorgung der Patient:innen aus Ihrer Sicht aussehen, wenn wirtschaftliche Faktoren keine Rolle spielen würden?

**Telemedizinische Aspekte**	Innerhalb des GeRas Projektes wurden telemedizinische Anwendungen integriert…<br><br>… welche Erfahrungen haben Sie bereits mit dem Einsatz von telemedizinischen Anwendungen bei geriatrischen Patient:innen gemacht?	• Welche Erwartungen haben Sie an ein Nachsorgeprogramm mit telemedizinischen Aspekten?<br>• Was gilt es Ihrer Meinung nach beim Einsatz telemedizinscher Anwendungen bei einer geriatrischen Zielgruppe zu beachten?<br>○ Welche Besonderheiten sehen Sie?<br>• Welche Vor- und Nachteile sehen Sie in der Nutzung telemedizinischer Anwendungen?<br>○ Speziell bei geriatrischen Patient:innen?
**Wirksamkeit**	Was kann ein Nachsorgeprogramm wie GeRas Ihrer Ansicht nach zu einer gelingenden Nachsorge beitragen?	• Für welche Patientengruppen eignet sich Ihrer Meinung nach ein solches Nachsorgeprogramm?<br>○ Und für welche vielleicht eher weniger?
	Inwiefern wünschen Sie sich Unterstützung in der Begleitung geriatrischer Patient:innen, wenn diese aus der stationären Behandlung in das häusliche Umfeld entlassen werden?	• Wie könnte diese Unterstützung Ihrer Meinung nach gestaltet werden?
**Skalierbarkeit**	GeRas wurde für die post stationäre Rehabilitation entwickelt.<br><br>Könnten Sie sich eine Ausweitung auf andere geriatrische Settings wie geriatrische Komplexbehandlungen in der Akutgeriatrie vorstellen?	• Worauf würden Sie bei einer deutschlandweiten Einführung eines Nachsorgeprogrammes wie GeRas besonders achten?
**Abschlussfrage**	• Wir haben jetzt über sehr viele Dinge gesprochen, was haben wir vielleicht noch nicht angesprochen? Was wäre Ihnen noch wichtig zu erwähnen?	

English Version

Theme	Main Questions	Follow-Up Questions
Opening Question	**You have already received information about the GeRas project. The aim of the GeRas project is to implement an aftercare program using tablets or posters as part of geriatric rehabilitation. Roughly speaking, the aim is to offer geriatric patients a program after inpatient rehabilitation that is designed to facilitate the transition from inpatient rehabilitation to the home environment and consolidate the training successes achieved during inpatient rehabilitation. It is a 12-week aftercare program with various components. It is aimed at geriatric patients who are discharged to their home environment.**<br>**Are you already familiar with a similar aftercare program, or is there something similar in your area?**	
**Experiences with the clientele**	Based on your experience, how is the transition between inpatient rehabilitation treatment and the home environment currently structured?	• How do you currently care for geriatric patients following inpatient rehabilitation or complex geriatric treatment?<br>• What experience do you have with rehabilitation aftercare programs?<br>• What situations do you encounter in your daily work with geriatric patients after an inpatient stay?
**Expectations for the aftercare program**	What are your expectations of an aftercare program such as GeRas?	• How would you design an aftercare program for geriatric patients?<br>○ What would be particularly important to you?<br>• In your experience, what conditions must be in place for an aftercare program for geriatric patients to work?<br>• In your experience, what conditions must be in place for an aftercare program for healthcare workers to work?<br>• What role do you see for your profession in an aftercare program?<br>• Which different key stakeholders should be involved in designing the aftercare program?<br>• How do you rate the outpatient aftercare currently provided as part of standard care following geriatric rehabilitation measures?<br>○ What would you consider to be the optimal care for patients if economic factors were not a consideration?

**Telemedicine aspects**	Telemedicine has been integrated into the GeRas project...<br><br>...what experience have you had with the use of telemedicine in geriatric patients?	• What are your expectations of an aftercare program with telemedicine aspects?<br>• In your opinion, what needs to be considered when using telemedicine applications for a geriatric target group?<br>• What special features do you see?<br>• What advantages and disadvantages do you see in the use of telemedicine applications?<br>○ Specifically for geriatric patients?
**Effectiveness**	In your opinion, how can an aftercare program such as GeRas contribute to successful aftercare?	• In your opinion, for which patient groups is such an aftercare program suitable?<br>○ And for which groups is it perhaps less suitable?
	To what extent would you like support in accompanying geriatric patients when they are discharged from inpatient treatment to their home environment?	• How do you think this support could be structured?
**Scalability**	GeRas was developed for post-hospital rehabilitation.<br><br>Could you imagine extending its use to other geriatric settings?	• What would you pay particular attention to if a follow-up care program such as GeRas were to be introduced throughout Germany?
**Closing Question**	We have now discussed many things, but what might we not have addressed yet? What else would be important for you to mention?	
