# Supplementary material for: Requirements for the scale up of a geriatric aftercare program: a qualitative interview study with stakeholders – findings from the GeRas project
Source: BMC Geriatr. 2026 Apr 30;26:612. doi: 10.1186/s12877-026-07567-8 (PMC13130819; doi:10.1186/s12877-026-07567-8)
Supplement: Supplementary file 3 — Supplementary Material 3. [file 12877_2026_7567_MOESM3_ESM.pdf]

Results of the Content Analysis

	Theme	Definition	Subthemes
1	Perception of Current Care after Discharge from the Inpatient to Outpatient Geriatric Care	The experiences of stakeholders after being discharged from an inpatient geriatric setting and transitioning to outpatient care are the focus of this theme.	• Patients' Fear of Discharge<br>• Perception of Inadequate Care Structures<br>• Non-Selective Distribution of Aftercare Approaches
2	Requirements for the Scalability of a multimodal, home-based Geriatric Aftercare Program	This theme reflects the views and expectations of diverse stakeholders in Germany concerning the requirements for scaling up and sustaining of geriatric aftercare programs in general.	• Structural Requirements<br>• Educational Concept<br>• Personnel Requirements and Network Building<br>• Financial Requirements and Remuneration<br>• Requirements for the Use of Telemedicine<br>• Key Requirements of a Geriatric Aftercare Program
3	Aims of Geriatric Aftercare Programs	This theme describes the aims of a program after discharge in general.	• Relieving the Burden on the Healthcare System<br>• Prevention, Rehabilitation and Health Promotion
4	Requirements for the Target Group	This theme captures the criteria that the target group must meet to participate in an aftercare program	• Mental and Cognitive Conditions<br>• Physical Health Requirements<br>• Voluntariness
